# Diseases of the Intestines

**Published:** 1906-03-10

**Authors:** 


### DISEASES OF THE INTESTINES.

(*Continued from page* 370.)

**Ulcerative Colitis.**—In an interesting paper on this condition Murrell[2] comments on the increase in the number of cases which appears recently to have occurred. The symptoms and ætiology present a marked contrast to the membranous form; it is commoner in men than in women, pain and tenesmus are not prominent, the appetite is unimpaired, the anæmia is of a low grade, and the temperature is usually persistently raised. There is no mental depression. Copious diarrhœa, loss of weight and energy, and bed sores are the remaining symptoms, and the duration of the disease is not more than three or four years. Thus in almost every point it is in marked contrast to membranous colitis. Murrell describes two well-marked cases, and states that he has seen four others in which the only treatment which succeeded was high irrigation with 2 per cent. solution of argyrol, diluted with an equal part of boiling water. This gave uniformly good results, and Murrell advises that it should be preceded by an enema of borax. An interesting fact observed in one case was that four minutes after the enema was begun the patient vomited a liquid which was undoubtedly a solution of argyrol. Murrell gives a curious list of other known instances of rapid reverse peristalsis.

**Diarrhœa.**—A convenient classification has been suggested[3] for this symptom, according as it appears to be primary or secondary. In the latter class may be placed the diarrhœa accompanying acute infectious fevers, ulceration of the intestine, profound anæmias, Addison's disease, Bright's disease, passive catarrh due to congestion of the mucous membrane, and lardaceous disease. In the former class are included diarrhœa from errors in diet, sudden chills, sudden rise in atmospheric temperature, local irritants, such as drugs, bad milk, and tinned foods, abnormalities in biliary and pancreatic secretion, and nervous disturbances. This division is certainly practically convenient, but we would suggest that the elevation of atmospheric temperature should be omitted, as this in all probability is in many cases a remote cause, and merely acts through changes provided in foodstuffs, and that the diarrhœa produced by biliary and pancreatic disease should more logically be transferred to the "secondary" class. Practically it amounts to this, that the primary diarrhœas are all due to temporary disturbances, the cause of which is transient, and may be removed, while in the secondary class the diarrhœa is either a symptom of some gross lesion in the bowel, of a general intoxication of a grave character, or to the operation of mechanical causes outside the bowel itself. Catarrhal inflammation of the small intestine is distinguished by colicky pain, less frequent and copious motions, the presence of undigested food, and the absence of mucus from the stools. Catarrh of the large intestine may cause pain and tenderness over the colon, much mucus in the stools, and much tenesmus. As to treatment, rest and warmth and a purge, such as Hydrargyrum c. Cretâ or castor oil, are the first measures, if the cause is to be removed. When it cannot be removed it may be checked by bismuth subnitrate in 20-grain doses, or a combination of Bismuthi subchloridum, pulv. cretæ aromat., Sodii bicarb., and Spiritus ammon. aromat. Astringents such as kino and sulphuric acid, or sedatives such as opium, are useful after the irritant has been removed. Milk and soda water, weak cereals, and egg water are suitable diets. No hot liquids should be given, and collapse may require alcohol in some form. The writer very properly remarks that these must be given cold and in small doses. In cholera infantum stomach washing and high irrigation of the bowel should be practised, with hypodermic injections of normal salt solution for collapsed cases.

[2] Birmingham Med. Rec., June, 1905, p. 338. [3] Practitioner, July, 1905, p. 122.

